# Comparison Between Presence of Epstein Barr Virus in Nodal and Extra Nodal Diffuse Large B Cell Lymphoma of Head and Neck, an Iranian Experience

**DOI:** 10.5812/ircmj.1302

**Published:** 2012-12-06

**Authors:** Mohammad Javad Ashraf, Alireza Makarempour, Ahmad Monabati, Negar Azarpira, Bijan Khademi, Afsoon Hakimzadeh, Elham Abedi, Bita Valibeigi

**Affiliations:** 1 Transplant Research Center, Department of Pathology, Shiraz Medical School, Shiraz University of Medical Sciences, Shiraz, IR Iran; 2 Department of Otolaryngology, Shiraz Medical School, Shiraz University of Medical Sciences, Shiraz, IR Iran

**Keywords:** Epstein-Barr Virus Infections, Lymphoma, Non-Hodgkin, Antigens, Head and Neck

## Abstract

**Background:**

Epstein Barr Virus (EBV) is one of the most common viral infections in human population. EBV has a significant role in pathogenesis of Hodgkin's lymphoma, Burkitt's lymphoma and nasopharyngeal carcinoma. The role of EBV in non-Hodgkin’s lymphoma, diffuse large B cell (NHL - DLBL) in the head and neck is controversial.

**Objectives:**

The purpose of this study is to find out the difference between the presence of Epstein Barr virus in nodal and extra nodal lymphoma of head and neck.

**Patients and Methods:**

A total of 30 cases of DLBL in two separate groups were collected from pathology department. The first group was consisted of 15 patients with DLBL of neck lymph node and the other was consisted of 15 patients with extra nodal DLBL of head and neck mainly in palatine tonsil. Both immune-histo-chemical (IHC) study and polymerase chain reaction (PCR) for detection of late membrane antigen (LMP) were performed on formalin fixed paraffin embedded tissue.

**Results:**

All 30 cases were negative for EBV in IHC method. But in PCR method, 10% of patients were positive for LMP gene. There were 2 positive cases in nodal lymphoma and 1 positive case in extra nodal lymphoma group.

**Conclusions:**

Compare with PCR method, it seems that IHC is not a sensitive method for detection of EBV. Overall, the finding of EBV in NHL depends on site, type of lymphoma and the detection method.

## 1. Background

Epstein bar virus (EBV) or human herpes virus 4 (HHV4), belongs to DSDNA virus from herpesviridae family, is a worldwide viral infection. EBV infects nearly all humans and persists for the life of the host. EBV was first isolated in 1964 by Epstein et al. (1) from African Burkitt's lymphoma cell lines and estimated prevalence in the total human populations of 80-90 %. Close personal contact is usually required for transmission “kissing disease”.

In developing countries, the infection occurs at a much earlier age so that 90% of children over the age of 2 are seropositive. The virus infects squamous epithelial cells as well as B lymphocytes via the complement receptor, CR2 (CD21). EBV contains receptors identical to C3d receptors.It can induce infectious mononucleosis, Hodgkin's lymphoma, Burkitt's lymphoma, nasopharyngeal carcinoma and post-transplantlympho-proliferative disease. It has Also a role in chronic fatigue syndrome, dermatomyositis and multiple sclerosis. EBV has also been linked to a variety of other epithelial and lymphoid derived proliferative diseases, including thymic carcinoma (2), gastric carcinoma (3), oral hairy leukoplakia, non-Hodgkin’s lymphoma of B cell origin (3) and Hodgkin's disease occurring in immune compromised patients. Generally, the rate of EBV infection in Hodgkin's lymphoma is higher than non-Hodgkin’s lymphoma (4, 5). The possible mechanism of EBV role in inducing lymphoma is interfering with normal function of P53 that regulate normal apoptosis, so it can lead to sustained genetic damage. Non-Hodgkin’s lymphoma (NHL) is a malignancy that arises from lymphoid tissue. In the United States, NHL is the fifth most commonly diagnosed cancer. About 30-40% of all malignant lymphoma arise primarily from extranodal sites. The most frequent sites of extra nodal lymphoma are gastrointestinal tract and the upper aero digestive tract (6). This disease is more common in men than in women and affects whites more often than African Americans or Asian Americans (7). NHL can occur at all age groups, however, it is more frequent in the elderly than in younger patients. The incidence of T-cell NHL is higher in Far East Asia countries than in Western and developed countries (8). Extra nodal head and neck lymphoma show a wide variability in morphology, anatomic location, the rate of EBV infection and prognosis depend on geographic places. For example NHL of Sino nasal tract is higher in south East Asian countries, most of them are NK/T cell variant, express CD56 in immune-phenotyping study and distinctly have an aggressive behavior and higher rate of EBV infection in compare with the same lymphoma in western countries (9). These variations among different populations suggest an interaction of environmental factors such as EBV as an oncogenic virus (10). NHL is more common among people who have abnormal or compromised immune systems, such as those with inherited immune deficiencies, individuals with autoimmune disorders, AIDS and people taking immunosuppressant drugs following organ transplants (5, 11). EBV is associated with post-transplantlympho proliferative disorders (PTLD) (11). Drinking water contaminated with nitrate and smoking may increase the risk of NHL (12, 13). Diffuse large B cell lymphoma (DLBL) is one the most common sub type of NHL worldwide. The disease frequently presents as a rapidly enlarging lymph node in 50%. The site is extra nodal in up to 40% of affected patients. In practice, DLBL is diagnosed by morphology and positivity for CD20 (mature B-cell marker) and CD79a (pan–B-cell marker). CD10 is expressed in 25-50% of cases and CD5 is expressed in a small proportion (14, 15). The proliferation index, measured by Ki-67 expression, is usually higher than 40% to 50%, and values greater than 80% may be associated with a worse outcome (16).

## 2. Objectives

The purpose of this study is to find out the difference between the presence of Epstein Barr virus in nodal and extra nodal lymphoma of head and neck.

## 3. Patients and Methods

**Figure 1:**
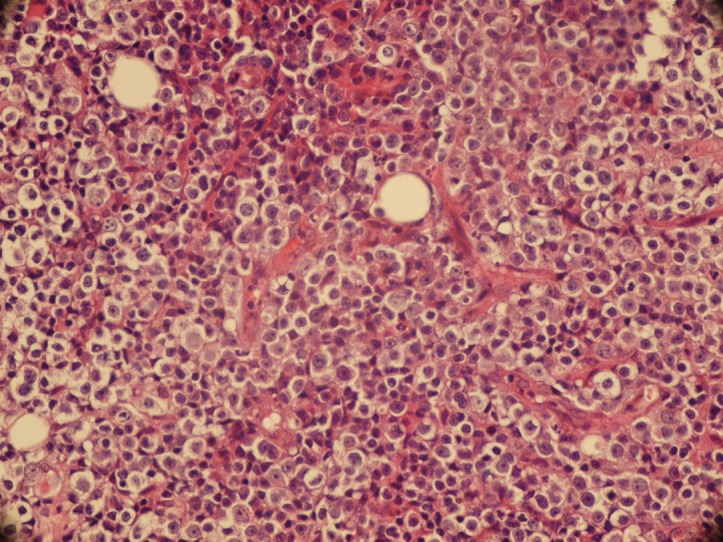
Sheet of DiscohesiveLarge Tumor Cell Infiltrated the Normal Parenchyma (H&E × 200)

**Figure 2:**
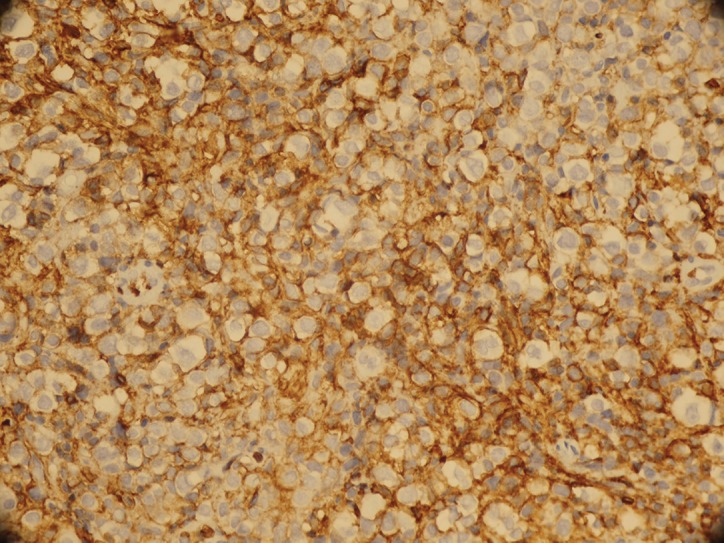
Positive Immunostaining for CD 20 (IHC× 200)

A total of 30 cases of lymphomas (DLBL) in head and neck region were collected from pathology departments of Khalili, Faghihi and Nemazi hospital, affiliated to Shiraz University of Medical Sciences, from 2003 to 2009. None of them had history of immunosuppression. The study was approved by the ethics committee of Shiraz University of Medical Sciences. All patients were from the same geographic region (southern Iran). The slides and reports were reviewed by 3 independent pathologists. Among them, 15 cases belonged to category of nodal lymphoma (NL-NHL) and 15 cases belonged to extra nodal lymphoma of head and neck region (EN-NHL) (Figures 1, 2). The immunohistochemical (IHC) markers which were used for diagnosing, composed of leukocyte common antigen (LCA, CD45), CD20 and CD79 (as a common B cell marker), CD3 (as a T cell marker), Ki67 (MIB-1), (as a proliferative index) and cytokeratin (CK) as an epithelial marker for rule out of metastasis. All primary antibodies were purchased from Dako, Denmark. Envision Dual link system-HRP (ready to use, Dako) was used as the secondary antibody. Incubation with 3,3-diaminobenzidine tetrahydrochloride as a chromogen substrate solution produced brown color. IHC and polymerase chain reaction (PCR) were used as two different methods for detection of EBV in paraffin sections. Latent membrane protein -1 (LMP-1, DAKO Denmark) was used as primary antibody for detection of EBV. For PCR, DNA was extracted manually by using xylol (for deparafinising), serial dilutions of ethanol (for tissue rehydration) and lysis buffer (EDTA, SDS10%, Proteinase K and Tris HCL 1M). The β- gloubin was used as internal control.

Forward primer: 5' CCA GAC AGC AGC CAA TTG TC 3'

Reverse primer: 5' GGT AGA AGA CCC CCT CTT AC 3'

(MOLBIOL company, Berlin) were used for amplification of 129 bp of LMP-1 gene.The region was amplified with a Mastercycler (Eppendorff, Germany), (35 cycles of 94 °C for 60 s, 60 °C for 60 s, and 72 °C for 1 min), in a 25 μl reaction solution containing 0.5 μg genomic DNA, 1× PCR buffer, 0.3 mM MgCl2, 0.3 mMdNTPs, 2 U Taq DNA polymerase (Cinagene, Iran) and 0.3 μmol of each primer.

## 4. Result

All of the patients were Iranian including 16 men and 14 women, with mean average age of 53.5 years (Range from 9 to 81 years). The ratio of male to female was 8 to 7. By considering group, in nodal lymphoma there were 9 men and 6 women with mean average ages of 51 years (Range 9- 80 years). In extra nodal group, 7 men and 8 women with mean average ages of 56 years (Ranges 16- 81 years) were included. The clinical presentation of nodal lymphoma was neck mass and another group presented with symptoms like dysphasia or hearing loss. The primary site of all nodal lymphoma was cervical lymph nodes; where in extra nodal group, palatine tonsil and Sino nasal region were the primary site of 12 and 3 patients respectively. The tumor cells were positive for CD20 (Figures 1 and 2), CD79 and CD45 in large cells and negative for CK and CD3. The range of Ki67 positivity was 20-90%. In IHC, all patients were negative LMP-1 antigen. By using PCR method to amplify the LMP gene of EBV, we had 3 (10%) positive cases. Two cases belonged to nodal lymphoma and 1 case to extra nodal group (Tables 1, 2).

**Table 1:** Results of EBV detection in nodal lymphoma group

Case	Age	Sex	Site	Type	Ki67	Ki67 score	PCR^a^	IHC ^a^
**1**	17	F	NECK L.N^a^	DLBL^a^	90%	4	─	─
**2**	56	F	NECK L.N	DLBL	30%	2	─	─
**3**	77	M	NECK L.N	DLBL	90%	4	─	─
**4**	11	M	NECK L.N	DLBL	20%	2	─	─
**5**	42	M	NECK L.N	DLBL	40%	3	─	─
**6**	80	F	NECK L.N	DLBL	80%	4	─	─
**7**	76	M	NECK L.N	DLBL	40%	3	─	─
8	71	F	NECK L.N	DLBL	80%	4	+	─
9	72	F	NECK L.N	DLBL	70%	4	─	─
10	46	M	NECK L.N	DLBL	60%	3	─	─
12	23	M	NECK L.N	DLBL	70%	4	+	─
13	55	M	NECK L.N	DLBL	80%	4	─	─
14	74	F	NECK L.N	DLBL	70%	4	─	─
15	9	M	NECK L.N	DLBL	90%	4	─	─

^a^Abbreviations: L.N: Lymph node, DLBL: Diffuse Large B cell Lymphoma, IHC: Immunohistochemical Study, PCR: Polymerase Chain Reaction

**Table 2:** Results of EBV Detection in Extra Nodal Lymphoma Group

Case	Age	Sex	Site	Type	Ki67	Ki67 score	PCR^a^	IHC^a^
**1**	64	F	sinonasal	DLBL^a^	80%	4	─	─
**2**	40	F	Palatine tonsil	DLBL	40%	3	─	─
**3**	76	M	Palatine tonsil	DLBL	20%	2	─	─
**4**	65	F	Palatine tonsil	DLBL	30%	2	+	─
**5**	50	M	Palatine tonsil	DLBL	60%	3	─	─
**6**	32	M	Palatine tonsil	DLBL	80%	4	─	─
**7**	66	F	Palatine tonsil	DLBL	70%	4	─	─
**8**	74	F	Palatine tonsil	DLBL	80%	4	─	─
**9**	80	F	Palatine tonsil	DLBL	80%	4	─	─
**10**	70	M	Palatine tonsil	DLBL	70%	4	─	─
**12**	56	F	Palatine tonsil	DLBL	90%	4	─	─
**13**	16	M	sinonasal	DLBL	10%	1	─	─
**14**	23	M	sinonasal	DLBL	70%	4	─	─
**15**	41	M	Palatine tonsil	DLBL	90%	4	─	─

^a^Abbreviations: DLBL: Diffuse large B cell lymphoma, IHC: Immunohistochemical study, PCR: Polymerase Chain Reaction

## 5. Discussion

EBV has been associated with a number of malignant lymphoma including Hodgkin, non-Hodgkin, Burkitt's and post-transplant B-cell lymphoma. Although most of these lymphomas have B-cell origin, the EBV genome has been detected in T-cell lymphomas especially when it originates from nasal cavity (17, 18). The role of EBV in pathogenesis of NHL was evaluated in different parts of the world (Table 3). The rate of EBV positivity and its role as an etiology is dependent to 5 major factors:

**Table 3:** Comparison of Some Related Published Articles

Reference	Year	Country	Site	Subtype	Number	Method	Result (% positive for EBV)
Ko et al. (31)	2004	Korea	ENNHL a	TCL a	11	ISH- EBER a	54%
						PCR	0%
Mitarnun et al. (24)	2006	Thailand	Sino nasal	89%TCL	16	ISH - EBER	100%
				11%BCL	2		0%
			Nasopharynx	17%TCL	7		100%
				83%BCL	35		5.4%
Feng et al. (33)	2007	China	Sino nasal	DLBL a	6	PCR and ISH For all cases	0%
				NKC a /T cell	44		100%
Tai et al. (6)	2004	Thailand	Sino nasal	NKC/T cell	20	PCR	100%
Quintanilla-Martinez et al. (25)	1999	Peru	Sino nasal	NKC/T cell	28	ISH-EBER	90%
van de Rijn et al. (23)	1997	Guatemala	Sino nasal	Head and neck	17	ISH-EBER	38%
Bahnassy et al (35)	2006	Egypt	EN-NHL head and neck	62% B-cell	50	PCR	70%
				18% T-cell		ISH-EBER	90%
				20% Nk-cell			
Ko et al. (31)	1994	Korea	NHL	TCL	50	ISH-EBER	61%
				BCL a			24%
Calzolari et al. (19)	1998	Italy	Oral NHL (HIV Patients)	DLBL	6	ISH-EBER	100%
Leong et al. (21)	2001	Canada	Oral NHL	All subtypes	9 Immuno compramised	ISH-EBER	100%
						IHC-LMP	62%
					46 Immuno competent	ISH-EBER	34%
						IHC-LMP	10%
				DLBL	22 Immuno compramis	ISH-EBER	27%
						IHC-LMP	9%
					3 Immuno competent	ISH-EBER	100%
						IHC-LMP	100%
Mitarnun et al. (24)	2006	Thailand	NHL	BCL	100	ISH-EBER	13%
				TCL	100	ISH-EBER	51%
				CHL	100	ISH-EBER	64%
Takahara et al. (32)	2004	Japan	NHL	NK/TCL	32	ISH-EBER	96%
						PCR	48%

^a^Abbreviations: TCL: T- cell lymphoma, BCL: B cell lymphoma, NKC: Natural killer cell, EN-NHL: Extra Nodal Non-Hodgkin’s lymphoma, DLBL: Diffuse large B cell lymphoma, ISH-EBER: In Situ Hybridization -EBV (Epstein-Bar Virus)-Encoded RNA (Ribonucleic Acid)

1. The state of patient's immunity (Immunocompromised or immunocompetent)

2. The method of EBV Detection (IHC, PCR and ISH-EBER)

3. The type of lymphoma (Hodgkin's versus non-Hodgkin’s) and Lymphoma subtypes (B-cell, T-cell, NK-cell, MALTOMA, etc.)

4. The anatomic site of lymphoma (Sino-nasal, head and neck, gastro intestinal tract, etc)

5-The geographic region of study (South East Asia versus Europe and North America)

### 5.1. State of Immunity

In 1999, Calzolari et al. (19) demonstrated that EBV infection is highly associated with NHL of the head and neck in HIV-infected patients. Their data suggested that LMP-1 expression may cause p53 loss of function even in the absence of p53 gene mutations, as assessed by SSCP. Similarly, Leong et al. (20) reported that all immunosuppressed patients with large B-cell oral lymphoma had EBV infection in compare with only 9% of immunocompetent patients. In 2001, Leong et al. (21) showed the strong concordance between EBV infection and NHL of oral cavity in immunosuppressed patients, all of the 9 immunosuppressed patients were positive for EBV (100%) whereas only 34% of immunocompetent patients were positive for EBV with ISH-EBER method. (21). In 2004, Iamproon et al. (19) in Thailand studied on 5 cases of immunosuppressed and 6 cases of immune competent NHL and showed all of immunosuppressed patients were positive for EBV by ISH method whereas only 40% of immune competent patients were positive for EBV. These studies are in agreement with our study. The result of our study on 30 immuno competent patients with lymphoma show only 13% EBV positive in nodal lymphoma and 7% EBV positive in extranodal lymphoma by PCR method.

### 5.2. Method of EBV Detection

In 1997, Van de Rijn in Guatemala studied on 17 cases of Sino nasal lymphoma and 16 cases of non-Sino nasal lymphoma in head and neck region by two methods of IHC and ISH-EBER (22). They showed significant difference in results depend to the method. (5% in IHC method and 38% in EBV (Epstein-Bar Virus)-Encoded RNA (Ribonucleic Acid). In Situ Hybridization, (EBER-ISH) method). The study is in agreement with our study that shows IHC method as an unreliable method for detecting EBV in NHL of head and neck. Mitarnun in Thailand studied on 60 cases of extranodal lymphoma and found that, ISH-EBER was a sensitive method for detection of EBV (23). The similar study with the same results was done by Martinez et al. (24) in Peru (1999). Bahnassy et al. (25) studied on 50 cases of EN-NHL of head and neck in Egypt. They used two methods of EBV detection e.g; PCR and ISH-EBER. The positive results with PCR and EBER-ISH were 70% and 90% respectively.

They explained although both methods were highly sensitive techniques but the difference between the results was attributed to difficulty in obtaining good-quality amplifiable DNA from paraffin-embedded tissues, heterogeneity of tumors and the ratio of tumor/normal cells. In another study more than 95% concordance was detected between PCR results and EBER-ISH for detection of EBV (26, 27). Leong et al. (21, 28) suggested that the EBER-ISH method was the most sensitive method for EBV detection. The sensitivity of PCR for EBV detection depends on part of EBV genomes which chose as a target for amplification. It is supposed that the most sensitive gene is BamH1W (21).

### 5.3. The Type of Lymphoma

Ko et al. (29) found that 54% of T-cell lymphoma (TCL) was positive in Korea. Mitranun (23) in Thailand showed all of the 19 cases of TCL were positive for EBV by ISH-EBER method. The similar results were seen in Tai et al. (24) study in Thailand and Martinez et al. in Peru (6). Francesco D amore et al. (4) studied on 374 cases of non-Hodgkin’s lymphoma of DLBL type in Denmark. They showed that only 7% of cases were positive for EBV by RNA-ISH method. Mitranun et al. (16) studied on 35 cases of nasopharyngeal B-cell lymphoma in Thailand. They found only 5.4% positivity for EBV. They also studied on 300 cases of lymphoma (100 cases of BCL, 100 cases of TCL and 100 cases of CHL). The rate of EBV infection by EBER-ISH method was 13%, 51% and 64% respectively (30). Feng Y F et al. (31)studied on 6 cases of DLBL of Sino nasal lymphoma in China. All of them were negative for EBV (PCR and ISH). Takahara et al. (32) found the EBV-encoded small nuclear early region (EBER) RNA in 96% of tumors. Above mentioned studies revealed that EBV has more potent role in inducing T-cell and NK-cell lymphoma rather than B-cell lymphoma. This finding is in agreement with our study that shows 10% EBV positive in NHL- DLBL.

### 5.4. The Anatomic Site of Lymphoma

The Sino nasal region was the most common sites for EBV associated lymphoma in head and neck region in several studies. Mitranun et al. showed that all of the 16 cases of lymphoma in Sino nasal region were positive for EBV. The same result was obtained in another study by Feng et al. (23, 31) on 44 cases of Sino nasal lymphoma. Tai et al. (24) in Thailand and Martinez et al. (6) in Peru showed the high prevalence of EBV infection in Sino nasal lymphoma. 127 cases of lymphoma from different anatomical sites were studied by Yang et al. (33) and high prevalence of EBV infection was found in nasopharyngeal area.

### 5.5. The Geographic Region Which Study Were Done

The rate of EBV in lymphomatous tissue in a specific geographic region is dependent to the overall rate of EBV infection in that region. Bahnassy et al. (34) in 2006 in Egypt showed 90% EBV positive in their lymphoma of head and neck region. They explained this high frequency of EBV infection in extra nodal lymphoma could be due to relatively high frequency of EBV infection in normal Egyptian population, since they detected EBV DNA in 40% of their normal samples. There is high prevalence of EBV associated lymphoma in counties like China and Korea. The high prevalence of EBV infection in their populations can explain this concordance. (6, 23). Vannarat et al. (35) claimed that, the EBV strains distribution may be associated with geographic/ethnic and clinical outcomes in the Thai population. Certain EBV strains defined by their LMP1 sequence may influence cell tropism, disease association, or disease severity. It seems that IHC is not a sensitive method for detection of EBV as compare with PCR. The finding of EBV in NHL depends on site, type of lymphoma and detection method. The attribute factors such as heterogeneity of tumors, the ratio of tumor/normal cells and the presence of EBV particles in normal homing lymphocytes are attributed for different results, when using different methods.
